# Pharmacological Activation of Sirt1 Ameliorates Cisplatin-Induced Acute Kidney Injury by Suppressing Apoptosis, Oxidative Stress, and Inflammation in Mice

**DOI:** 10.3390/antiox8080322

**Published:** 2019-08-19

**Authors:** Jung-Yeon Kim, Jungmin Jo, Kiryeong Kim, Hyun-Jin An, Mi-Gyeong Gwon, Hyemin Gu, Hyun-Ju Kim, A Young Yang, Sung-Woo Kim, Eon Ju Jeon, Jae-Hyung Park, Jaechan Leem, Kwan-Kyu Park

**Affiliations:** 1 Department of Immunology, School of Medicine, Catholic University of Daegu, Daegu 42472, Korea; 2 Department of Hematology-Oncology, Inje University Seoul Paik Hospital, Seoul 04551, Korea; 3 Department of Physiology, School of Medicine, Keimyung University, Daegu 42601, Korea; 4 Department of Pathology, School of Medicine, Catholic University of Daegu, Daegu 42472, Korea; 5 Department of Internal Medicine, School of Medicine, Catholic University of Daegu, Daegu 42472, Korea

**Keywords:** sirtuin 1, SRT1720, cisplatin, acute kidney injury, apoptosis, oxidative stress, inflammation

## Abstract

Sirtuin 1 (Sirt1) is an essential modulator of cellular metabolism and has pleiotropic effects. It was recently reported that Sirt1 overexpression in kidney tubule ameliorates cisplatin-induced acute kidney injury (AKI). However, whether pharmacological activation of Sirt1 also has a beneficial effect against the disease remains unclear. In this study, we aimed to evaluate whether SRT1720, a potent and specific activator of Sirt1, could ameliorate cisplatin-induced AKI. We found that SRT1720 treatment ameliorated cisplatin-induced acute renal failure and histopathological alterations. Increased levels of tubular injury markers in kidneys were significantly attenuated by SRT1720. SRT1720 treatment also suppressed caspase-3 activation and apoptotic cell death. Increased expression of 4-hydroxynonenal, elevated malondialdehyde level, and decreased ratio of reduced glutathione/oxidized glutathione after cisplatin injection were significantly reversed by SRT1720. In addition, SRT1720 treatment decreased renal expression of pro-inflammatory cytokines and prevented macrophage infiltration into damaged kidneys. We also showed that the therapeutic effects of SRT1720 were associated with reduced acetylation of p53 and nuclear factor kappa-B p65 and preservation of peroxisome function, as evidenced by recovered expression of markers for number and function of peroxisome. These results suggest that Sirt1 activation by SRT1720 would be a useful therapeutic option for cisplatin-induced AKI.

## 1. Introduction

Cisplatin is a chemotherapy drug used for the treatment of many different types of cancer, including ovarian, testicular, bladder, and lung cancer [1,2,3]. However, approximately 25–30% of patients treated with cisplatin suffer from acute kidney injury (AKI), and thereby this complication limits its clinical use. Although some strategies including intensive hydration have been used, there is no satisfactory treatment to protect against cisplatin-induced AKI.

Sirtuin 1 (Sirt1) is a histone deacetylase implicated in regulating metabolic responses to nutrient availability [4]. Numerous studies suggest that Sirt1 is mainly responsible for modulation of energy metabolism in metabolic tissues [5]. In addition, Sirt1 is also known to inhibit cell apoptosis, inflammatory responses, and oxidative stress through deacetylating a variety of substrates including p53 [6,7] and p65 subunit of nuclear factor kappa-B (NF-κB) [8,9]. Thus, Sirt1 has been recognized as a useful therapeutic target for the therapy of numerous inflammatory diseases. It was shown that Sirt1 overexpression in kidney tubules ameliorates cisplatin-induced AKI by inhibiting apoptotic cell death and oxidative stress [10]. However, whether pharmacological activation of Sirt1 also has a beneficial effect against cisplatin-induced AKI has not yet been fully determined.

SRT1720 is a small molecule that potently and specifically activates Sirt1 [11]. Sirt1 activation by SRT1720 was shown to extend the lifespan of rodents [12] and to ameliorate diet-induced obesity and metabolic dysfunction [11,13]. Moreover, SRT1720 treatment suppressed ovalbumin-induced airway inflammation [14] and protected against cigarette smoke-induced lung injury [15]. Liver injury after hepatic ischemia-reperfusion [16] and multiorgan injury in sepsis [17] were also attenuated by SRT1720. In this study, we aimed to evaluate whether SRT1720 treatment could ameliorate against cisplatin-induced AKI.

## 2. Materials and Methods

### 2.1. Animals and Drug Treatment

Seven-week-old male C57BL/6N mice (Samtako, Daejeon, South Korea) were adapted to the facility for 1 week before study. The mice were randomly grouped into the following groups (n = 8 for each group): control (Con), cisplatin alone (CP), and SRT1720 in combination with cisplatin (CP+SRT1720). To induce AKI, mice were intraperitoneally injected with cisplatin (15 mg/kg in 0.9% normal saline). Control mice were intraperitoneally injected with an equal volume of the vehicle. To investigate the effects of SRT1720 (Santa Cruz Biotechnology, Santa Cruz, CA, USA) on cisplatin-induced AKI, mice were intraperitoneally injected with SRT1720 (20 mg/kg) [16,17] for 3 days. The treatment with SRT1720 started 1 h before cisplatin injection. Mice were sacrificed 72 h after cisplatin injection. All animal experiments were performed according to the animal protocols approved by the Institutional Animal Care and Use Committee of the Catholic University of Daegu (DCIAFCR-180809-13-Y).

### 2.2. Histology and Immunohistochemistry

Kidneys were rapidly removed from each mouse and then fixed in 4% phosphate-buffered paraformaldehyde. The tissues were embedded in paraffin and then thin sections were made from the paraffin blocks. The sections were incubated with hematoxylin and eosin (H&E) stain and periodic acid Schiff (PAS) stain. Images were captured using the NIKON A1+ confocal microscope (Nikon, Tokyo, Japan). The degree of tubular injury was scored as previously described [18]. For immunohistochemical staining, the kidney sections were probed with antibodies against kidney injury molecule-1 (Kim-1; Abcam, Cambridge, MA, USA), neutrophil gelatinase-associated lipocalin (NGAL; Santa Cruz Biotechnology), 4-hydroxynonenal (4-HNE; Abcam), or Galectin-3 (Abcam).

### 2.3. Evaluation of Renal Function and Oxidative Stress

Creatinine and blood urea nitrogen (BUN) levels in plasma, malondialdehyde (MDA) levels in kidney tissues, and reduced glutathione (GSH)/oxidized glutathione (GSSG) ratio in kidney tissues were analyzed using commercial kits according to the manufacturer’s instructions.

### 2.4. Western Blot Analysis

Protein was isolated from kidney tissues and loaded onto a gradient polyacrylamide gel. The resolved proteins were transferred onto a nitrocellulose membrane and the membrane was incubated with the following primary antibodies: anti-Kim-1 (Abcam), anti-cleaved caspase-3 (Cell Signaling, Danvers, MA, USA), anti-Bax (Santa Cruz Biotechnology), anti-cleaved poly(ADP-ribose) polymerase-1 (PARP1; Cell Signaling), anti-acetyl-p53 (Lys379; Cell signaling), anti-p53 (Cell Signaling), anti-interleukin-6 (IL-6; Abcam), anti-tumor necrosis factor-α (TNF-α; Abcam), anti-acetyl-NF-κB p65 (Lys310; Cell Signaling), anti-NF-κB p65 (Cell Signaling), and anti- glyceraldehyde 3-phosphate dehydrogenase (GAPDH; Cell Signaling) (Cell Signaling) antibody. The membrane was washed and incubated with horseradish peroxidase-conjugated secondary antibodies, and signals were detected using an enhanced chemiluminescence detection system (Thermo Fisher Scientific, Waltham, MA, USA). The protein expression levels were normalized against GAPDH.

### 2.5. Terminal Deoxynucleotidyl Transferase -Mediated Deoxyuridine Triphosphate Nick End Labeling (TUNEL) Staining

Apoptosis was examined in the kidney sections using the in situ Cell Death Detection Kit (Roche Diagnostics, Indianapolis, IN, USA), according to the manufacturer’s instructions. Briefly, the kidney sections were deparaffinized in xylene, rehydrated using descending grades of ethanol, and permeabilized for 30 min at room temperature with proteinase K in 10 mM Tris-HCl, pH 7.4–8. After washing, the sections were incubated in the TUNEL reaction mixture for 1 h at 37 °C. Nuclei were counterstained with 4′,6-diamidino-2-phenylindole (DAPI). Images were captured using the NIKON A1+ confocal microscope.TUNEL-stained apoptotic cells were counted in five randomly chosen fields (×200 magnification) per kidney.

### 2.6. Gene Expression Analysis

Total RNA extraction from kidney tissue was performed using TRIzol reagent and one microgram of each sample was reverse transcribed into cDNA by using oligo (dT)18 primers and the AccuPower RT Premix (Bioneer, Daejeon, South Korea) according to the manufacturer’s instructions. Quantitative real-time RT-PCR was performed using the Real-Time PCR 7500 system (Applied Biosystems, Foster city, CA, USA) and Power SYBR Green PCR Master Mix (Applied Biosystems). Sequences of specific primers are listed in Table 1. GAPDH was used to normalize the expression levels of the other genes.

### 2.7. Statistical Analysis

Data are represented as the mean ± standard error of the mean (SEM) and analyzed using one-way ANOVA followed by a post-hoc Bonferroni’s multiple comparison test. *p* ˂ 0.05 was considered statistically significant.

## 3. Results

### 3.1. SRT1720 Ameliorated Cisplatin-Induced AKI

**Figure 1:**
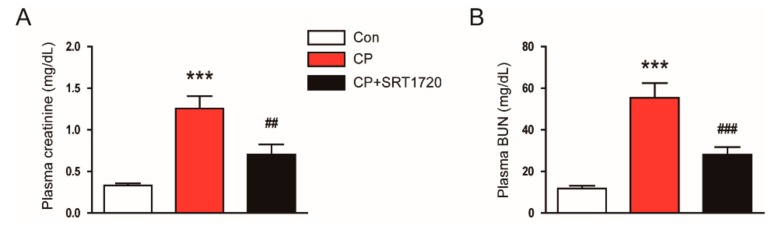
Effects of SRT1720 on renal function in mice treated with cisplatin. (**A**) Plasma creatinine. (**B**) Plasma blood urea nitrogen (BUN). *** *p* < 0.001 vs. control (Con). ^##^ *p* < 0.01 and ^###^ *p* < 0.001 vs. cisplatin alone (CP).

**Figure 2:**
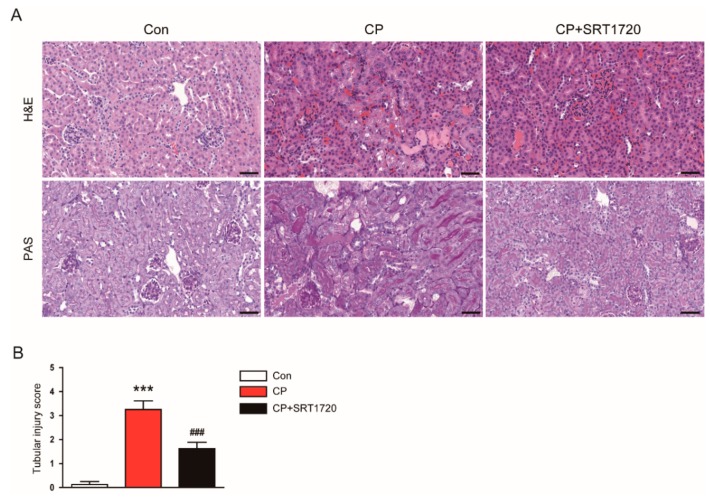
Effects of SRT1720 on renal histology in mice treated with cisplatin. (**A**) Representative images of hematoxylin and eosin (H&E) and periodic acid Schiff (PAS) staining. Scale bar: 50 μm. (**B**) Tubular injury score. *** *p* < 0.001 vs. Con. ^###^ *p* < 0.001 vs. CP.

Cisplatin-treated mice developed acute renal failure, as assessed by elevated levels of creatinine and BUN at 72 h after cisplatin injection (Figure 1A,B). The cisplatin-induced renal dysfunction was significantly attenuated by SRT1720. Histological staining of the kidney sections showed that cisplatin-treated mice displayed histopathological alterations such as dilated and cast-filled tubules (Figure 2A,B). The cisplatin-induced structural damages were also attenuated by SRT1720.

**Figure 3:**
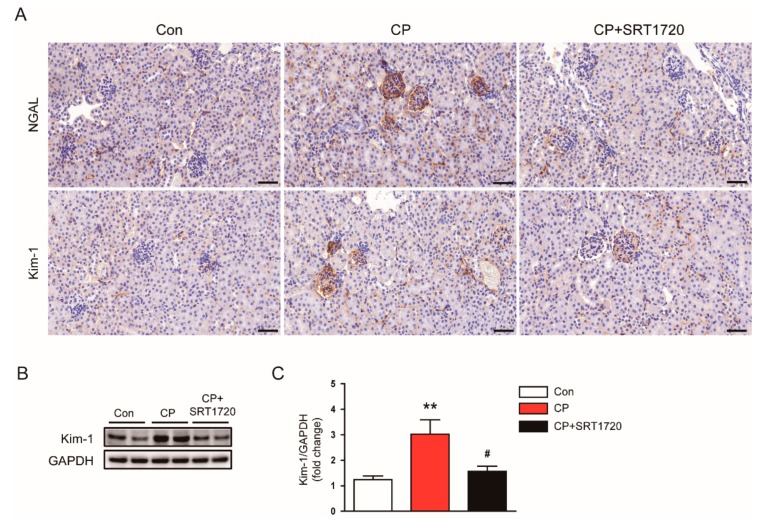
Effects of SRT1720 on renal expression of neutrophil gelatinase-associated lipocalin (NGAL) and kidney injury molecule-1 (Kim-1) in mice treated with cisplatin. (**A**) Representative images of immunohistochemical staining using anti-NGAL or anti-Kim-1 antibody. Scale bar: 50 μm. (**B**) Western blots of Kim-1 level in kidneys. (**C**) Quantification of Kim-1 level. ** *p* < 0.01 vs. Con. ^#^ *p* < 0.05 vs. CP.

To further investigate the effect of SRT1720 in tubule injury, we evaluated expression of NGAL and Kim-1 in kidneys. Immunohistochemical staining revealed that administration of SRT1720 reduced elevated level of NGAL and Kim-1 in damaged tubules of mice treated with cisplatin (Figure 3A). Consistently, the increased protein level of Kim-1 after cisplatin injection was markedly decreased by SRT1702 (Figure 3B,C).

### 3.2. SRT1720 Suppressed Cisplatin-Induced Cell Apoptosis

**Figure 4:**
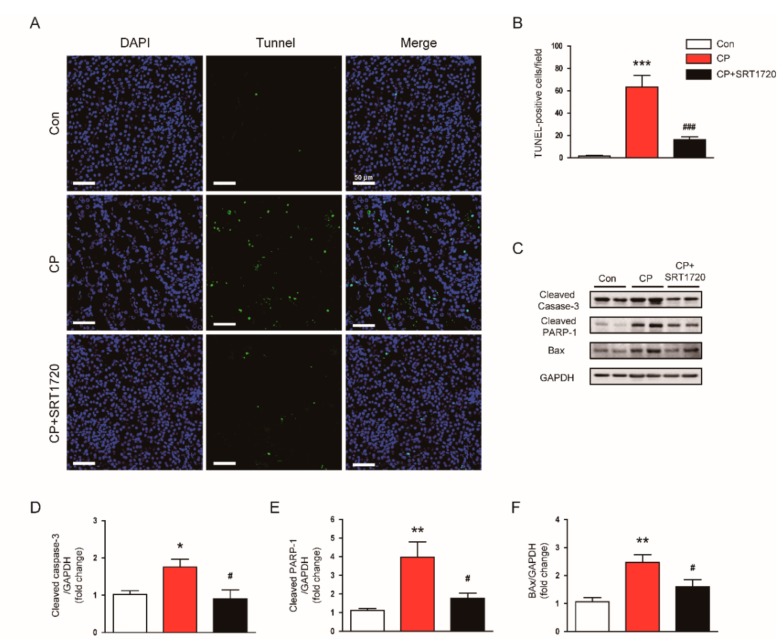
Effects of SRT1720 on apoptotic cell death in kidneys of mice treated with cisplatin. (**A**) Representative images of terminal deoxynucleotidyl transferase-tediated deoxyuridine triphosphate nick end labeling (TUNEL) staining. Scale bar: 50 μm. (**B**) Number of TUNEL-stained cells. (**C**) Western blots of cleaved caspase-3, cleaved poly(ADP-ribose) polymerase-1 (PARP1), and Bax levels in kidneys. (**D**) Quantification of cleaved caspase-3 level. (**E**) Quantification of cleaved PARP1 level. (**F**) Quantification of Bax level. * *p* < 0.05, ** *p* < 0.01, and *** *p* < 0.001 vs. Con. ^#^ *p* < 0.05 and ^###^ *p* < 0.001 vs. CP.

To explore the underlying mechanisms for the preventive actions of SRT1720 against cisplatin-induced AKI, we carried out TUNEL staining of the tissues to identify apoptotic cells. Administration of SRT1720 markedly decreased the number of TUNEL-stained apoptotic cells in kidney after cisplatin injection (Figure 4A,B). Moreover, the increased protein level of activated caspase-3, cleaved PARP1, and Bax in kidneys of mice treated with cisplatin was also significantly attenuated by SRT1720 (Figure 4C–F).

### 3.3. SRT1720 Suppressed p53 Acetylation in Mice Treated With Cisplatin

**Figure 5:**
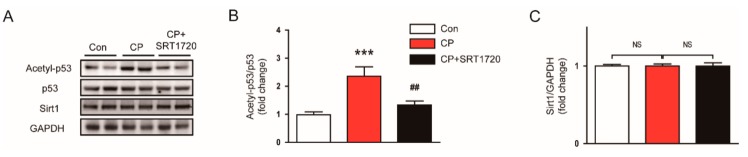
Effects of SRT1720 on Sirtuin 1 (Sirt1) expression and p53 acetylation in kidneys of cisplatin-treated mice. (**A**) Western blots of acetyl-p53, p53, and Srit1 levels in kidneys. (**B**) Quantification of acetyl-p53 level. (**C**) Quantification of Sirt1 level. *** *p* < 0.001 vs. Con. ^##^ *p* < 0.01 vs. CP. NS, not significant.

To obtain a more mechanistic insight into the effect of the Sirt1 activator, we examined protein levels of Srit1 and acetylated p53 (Lys379) in kidneys of SRT1720-treated mice. We observed that the acetylated p53 level was significantly elevated in kidneys of mice treated with cisplatin alone compared to control mice (Figure 5A,B), while the Sirt1 level was not changed (Figure 5A,C). SRT1720 treatment markedly suppressed the increased acetylation of p53, without affecting Sirt1 expression. Collectively, these findings suggest that Sirt1 activation by SRT1720 attenuates cisplatin-induced apoptotic cell death, presumably through deacetylating p53.

### 3.4. SRT1720 Decreased Cisplatin-Induced Oxidative Stress and Preserved Peroxisome Function

**Figure 6:**
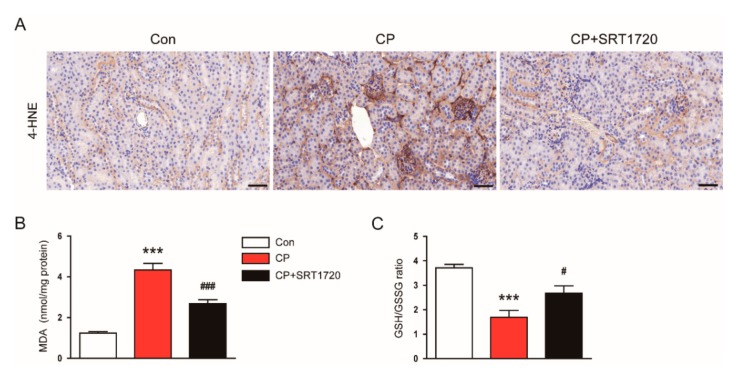
Effects of SRT1720 on renal oxidative damage in mice treated with cisplatin. (**A**) Representative images of immunohistochemical staining using anti-4-hydroxynonenal (4-HNE) antibody. Scale bar: 50 μm. (**B**) Renal malondialdehyde (MDA) level. (**C**) reduced glutathione (GSH)/oxidized glutathione (GSSG) ratio in kidneys. *** *p* < 0.001 vs. Con. ^#^ *p* < 0.05 and ^###^ *p* < 0.001 vs. CP.

We next explored the effect of SRT1720 on the renal oxidative stress induced by cisplatin. Immunohistochemical staining revealed that SRT1720 treatment significantly attenuated increased renal expression of 4-HNE in mice treated with cisplatin (Figure 6A). Moreover, SRT1720 significantly reversed elevated levels of MDA (Figure 6B) and reduced the GSH/GSSG ratio (Figure 6C) in kidneys of mice treated with cisplatin.

**Figure 7:**
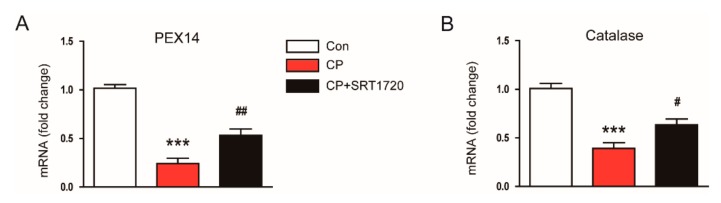
Effects of SRT1720 on markers of peroxisome number and function in mice treated with cisplatin. Real-time RT-PCR analysis of PEX14 (**A**) and catalase (**B**) in kidneys. *** *p* < 0.001 vs. Con. ^#^ *p* < 0.05 and ^##^ *p* < 0.01 vs. CP.

It has been shown that cisplatin reduces peroxisome number and impairs its function [19], and kidney tubule-specific overexpression of Sirt1 suppresses oxidative stress through preservation of peroxisome function [10]. Thus, we next evaluated the effect of SRT1720 on mRNA level of peroxisomal membrane protein 14 (PEX14), a marker for the number of peroxisomes, and level of catalase, a marker for peroxisome function. We found that mice treated with cisplatin alone exhibited a dramatic reduction in mRNA expression of PEX14 and catalase compared to control mice and these changes were largely reversed by SRT1720 (Figure 7A,B). Taken together, these results suggest that SRT1720 reduces cisplatin-associated oxidative stress, probably through preservation of peroxisome function.

### 3.5. SRT1720 Attenuated Cisplatin-Induced Inflammatory Responses

**Figure 8:**
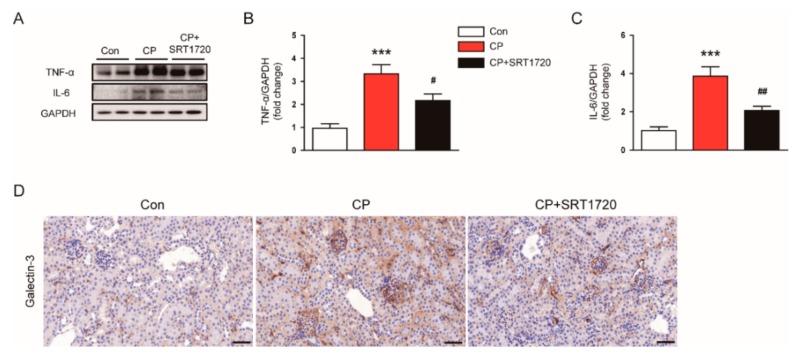
Effects of SRT1720 on renal inflammation in mice treated with cisplatin. (**A**) Western blots of tumor necrosis factor-α (TNF-α) and interleukin-6 (IL-6) levels in kidneys. (**B**) Quantification of TNF-α level. (**C**) Quantification of IL-6 level. (**D**) Representative images of immunohistochemical staining using anti-Galectin-3 antibody. *** *p* < 0.001 vs. Con. ^#^ *p* < 0.05 and ^##^ *p* < 0.01 vs. CP.

We next evaluated the effect of SRT1720 on cisplatin-induced inflammation. We observed that increased protein expression of TNF-α (Figure 8A,B) and IL-6 (Figure 8A,C) in kidneys of cisplatin-treated mice was greatly attenuated by SRT1720. To identify macrophages, kidney sections were probed with anti-Galectin-3 antibody. As shown in Figure 8D, administration of SRT1720 attenuated cisplatin-induced accumulation of Galectn-3-positive cells in kidneys.

### 3.6. SRT1720 Reduced Acetylation of NF-κB p65 In Mice Treated With Cisplatin

**Figure 9:**
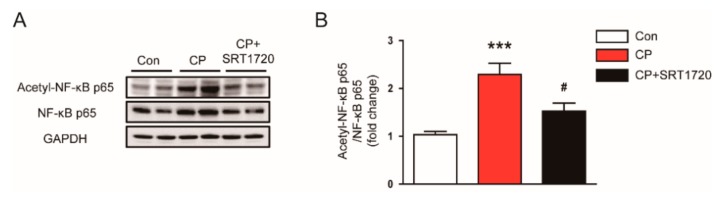
Effects of SRT1720 on acetylation of nuclear factor kappa-B (NF-κB) p65 in kidneys of mice treated with cisplatin. (**A**) Western blots of acetyl-NF-κB p65 and NF-κB p65 levels in kidneys. (**B**) Quantification of acetyl-NF-κB p65 level. *** *p* < 0.001 vs. Con. ^#^ *p* < 0.05 vs. CP.

Given that NF-κB is a key transcriptional factor for regulating inflammation, we next examined the effect of SRT1720 on NF-κB activation. We found that cisplatin markedly elevated acetylation of lysine 310 of NF-κB p65 (Figure 9A,B). Administration of SRT1720 significantly reduced the protein level of acetylated p65 in kidneys of mice treated with cisplatin. These findings suggest that Sirt1 activation of SRT1720 inhibits NF-κB, probably by deacetylating p65 subunit.

## 4. Discussion

In this study, we showed that Sirt1 activation by SRT1720 significantly attenuated acute renal failure and histopathological alterations in cisplatin-treated mice through suppression of apoptotic cell death, oxidative stress, and inflammation. These therapeutic effects of Sirt1 activation were associated with deacetylation of p53 and NF-κB p65 and preservation of peroxisome function. These findings reveal the preventive role of SRT1720 against cisplatin-induced AKI.

Acetylation, one of the post-translational modifications, is a molecular process of transferring acetyl groups. Deacetylation is its reverse reaction and is mediated by histone deacetylases [20]. Among them, Sirt1 can deacetylate numerous substrates involved in energy metabolism and aging [4,5]. It has been suggested that the histone deacetylase is also critically involved in the modulation of inflammatory responses [9]. Recently, it was reported that Sirt1 overexpression in renal tubules exerted protective effects against cisplatin-induced AKI [10]. Previous studies also showed that resveratrol, a naturally occurring Srit1 activator, ameliorates cisplatin-induced AKI [21,22]. However, given that resveratrol is not Sirt1-specific [23,24], these studies were not sufficient to clearly demonstrate the effect of pharmacological activation of Sirt1 against cisplatin-induced AKI. SRT1720 is a small molecule that potently and specifically activates Sirt1 [11]. In this study, we found that administration of SRT1720 ameliorates acute renal failure in cisplatin-treated mice, as represented by decreased levels of creatinine and BUN. Because cisplatin treatment induces structural damages in kidneys, especially tubular injuries [25,26,27], we further examined the effect of SRT1720 on levels of NGAL and Kim-1. We found that tubular injuries in kidneys of cisplatin-treated mice were also markedly prevented by SRT1720, as evidenced by reduced expression of NGAL and Kim-1. Taken together, these results strongly support the notion that Sirt1 would be a useful pharmacological target for cisplatin-induced AKI.

The mechanisms by which cisplatin causes AKI involve multiple processes. Among them, tubular cell apoptosis is considered an essential process in cisplatin-induced AKI [1,2,3]. In this study, administration of SRT1720 significantly inhibited caspase-3 activation and subsequent cleavage of PARP1 after cisplatin injection. Increased Bax expression in kidneys of cisplatin-treated mice was also significantly suppressed by SRT1720. Moreover, TUNEL assay confirmed the anti-apoptotic effect of SRT1720. In agreement with our findings, it was reported that tubule-specific overexpression of Sirt1 protected against cisplatin-induced AKI, and overexpression of Sirt1 in a cultured proximal tubule cell line prevented cisplatin-induced cell apoptosis [10].

The tumor suppressor p53 has a strong pro-apoptotic function and thereby its expression is tightly regulated in cells [6,7]. Normally, a low level of p53 protein is maintained via ubiquitination-dependent degradation. However, under stressful conditions, p53 protein is quickly accumulated and activated. Stabilization of p53 is controlled by its posttranslational modifications. Among them, acetylation enhances its site-specific DNA binding activity [28]. It has been shown that p53 plays a key role in cisplatin-associated renal injury [29,30]. Recently, cisplatin was reported to be able to increase acetylation of p53 at lysine 379 in renal tubular cells [31]. In this study, we observed that mice treated with cisplatin alone exhibited an increase in acetylation of p53 (Lys379) in kidneys. We also found that SRT1720 significantly reduced acetylation of p53 (Lys379) and the protein level of Bax, a transcriptional target of p53, in kidneys after cisplatin injection. Consistent with our findings, a previous study demonstrated that silencing of p53 by siRNA ameliorated cisplatin-induced apoptosis and histological damage in kidneys [32]. Collectively, these results suggest that Srit1 activation by SRT1720 attenuates cisplatin-induced cell apoptosis, probably through deacetylating p53.

Oxidative stress is also tightly associated with the development of cisplatin-induced AKI [1,2,3]. Furthermore, Sirt1 overexpression in renal tubules was shown to suppress cisplatin-induced oxidative stress through preventing peroxisomal dysfunction [10]. In this study, increased lipid peroxidation, as measured by an elevation both in 4-HNE and MDA levels, was significantly suppressed by SRT1720. SRT1720 also significantly reversed a reduction of the GSH/GSSG ratio in kidneys of mice treated with cisplatin. Moreover, SRT1720 significantly reversed cisplatin-induced reduction in mRNA expression of PEX14, a marker for the number of peroxisomes, and catalase, a marker for peroxisome function, in kidneys. Catalase, an enzyme with potent antioxidant properties, is mainly located in peroxisomes [33]. Previous studies reported the suppressive effect of cisplatin on catalase activity in mice kidney [34,35]. In addition, Sirt1 overexpression prevented oxidative stress-induced tubular cell apoptosis by increasing catalase level [36]. Catalase overexpression in renal tubules was shown to decrease oxidative stress and tubular cell apoptosis [37]. Collectively, these findings suggest that Sirt1 activation by SRT1720 attenuates cisplatin-induced oxidative stress, at least in part, through preservation of peroxisome function.

Inflammatory responses in kidney tissues are responsible for the development of cisplatin-induced AKI [1,2,3]. Among various mediators of renal injury, TNF-α is recognized as a critical mediator in the development of inflammation after cisplatin treatment. It was demonstrated that inhibition of TNF-α ameliorated renal dysfunction and histopathological alterations after cisplatin injection [38]. TNF-α has also been suggested as a key mediator in expression of pro-inflammatory cytokines and recruitment of immune cells into kidney tissues in cisplatin-induced AKI [39,40]. Although there remains some controversy, it has been suggested that macrophage infiltration into damaged kidneys is a crucial process in cisplatin-induced AKI [41]. In this study, cisplatin significantly increased TNF-α and IL-6 levels in kidneys. The number of galectin-3-positive macrophages was also increased after cisplatin injection. All these changes were significantly reversed by SRT1720. These results indicate that SRT1720 effectively suppresses cisplatin-induced inflammatory responses in kidneys. However, we think that SRT1720 also could directly affect immune cell activities to suppress cisplatin-induced renal inflammation, because previous studies have shown the regulatory role of Srit1 in the immune system [9].

NF-κB is a major transcription factor responsible for controlling expression of pro-inflammatory mediators. Thus, to obtain a more mechanistic insight into the effect of Sirt1 activation on inflammatory responses induced by cisplatin, we next evaluated the effect of SRT1720 on NF-κB signaling. We found that acetylation of NF-κB p65 (Lys310) was markedly increased in kidneys after cisplatin injection. This posttranslational modification was significantly reversed by SRT1720. These results are in agreement with a previous study that demonstrated the suppressive effect of Sirt1 overexpression on acetylation of NF-κB p65 at lysine 310 in cisplatin-treated renal tubular cells [42]. It was also demonstrated that Sirt1 binds with NF-κB p65 and deacetylates it, resulting in suppression of its transcriptional activity [8]. Collectively, our findings suggest that reduced acetylation of NF-κB p65 by SRT1720, at least in part, contributes to the preventive effect of SRT1720 against renal inflammation induced by cisplatin.

## 5. Conclusions

In conclusion, our data showed that Sirt1 activation by SRT1720 attenuates cisplatin-induced AKI by inhibiting apoptotic cell death, oxidative stress, and inflammation, probably via deacetylation of p53 and NF-κB p65 and preservation of peroxisome function. These results strengthen the idea that pharmacological activation of Sirt1 may be a promising therapeutic strategy against cisplatin-induced AKI.

## Figures and Tables

**Table 1:** Primers used for quantitative real-time RT-PCR.

Gene	Primer Sequence (5′→3′)	Product Size (bp)
PEX14 ^1^	Forward: GCCACCACATCAACCAACTG Reverse: GTCTCCGATTCAAAAGAAGTCCT	97
catalase	Forward: CAAGTACAACGCTGAGAAGCCTAAG Reverse: CCCTTCGCAGCCATGTG	74
GAPDH ^2^	Forward: ACTCCACTCACGGCAAATTC Reverse: TCTCCATGGTGGTGAAGACA	170

^1^ Peroxisomal membrane protein 14. ^2^ Gyceraldehyde 3-phosphate dehydrogenase.
